# Sentiment analysis of Indonesian tweets on COVID-19 and COVID-19 vaccinations

**DOI:** 10.12688/f1000research.130610.2

**Published:** 2023-11-22

**Authors:** Viskasari Pintoko Kalanjati, Nurina Hasanatuludhhiyah, Annette d'Arqom, Danial H. Arsyi, Ancah Caesarina Novi Marchianti, Azlin Muhammad, Diana Purwitasari

**Affiliations:** 1 Department of Anatomy, Histology and Pharmacology, Faculty of Medicine Universitas Airlangga, Surabaya, Indonesia; 2 Faculty of Medicine Universitas Airlangga, Surabaya, Indonesia; 3 Public Health Division, Faculty of Medicine Universitas Jember, Jember, Indonesia; 4 Faculty of Medicine, Universiti Kebangsaan Malaysia, Kuala Lumpur, Malaysia; 5 Department of Informatics, Institut Teknologi Sepuluh Nopember, Surabaya, Indonesia

**Keywords:** COVID-19, COVID-19 vaccination, Tweets, Sentiment analysis, Vaccine, social media

## Abstract

**Background:**

Sentiments and opinions regarding COVID-19 and the COVID-19 vaccination on Indonesian-language Twitter are scarcely reported in one comprehensive study, and thus were aimed at our study. We also analyzed fake news and facts, and Twitter engagement to understand people’s perceptions and beliefs that determine public health literacy.

**Methods:**

We collected 3,489,367 tweets data from January 2020 to August 2021. We analyzed factual and fake news using the string comparison method. The *difflib* library was used to measure similarity. The user’s engagement was analyzed by averaging the engagement metrics of tweets, retweets, favorites, replies, and posts shared with sentiments and opinions regarding COVID-19 and COVID-19 vaccination.

**Result:**

Positive sentiments on COVID-19 and COVID-19 vaccination dominated, however, the negative sentiments increased during the beginning of the implementation of restrictions on community activities (PPKM).  The tweets were dominated by the importance of health protocols (washing hands, keeping distance, and wearing masks). Several types of vaccines were on top of the word count in the vaccine subtopic. Acceptance of the vaccination increased during the studied period, and the fake news was overweighed by the facts. The tweets were dynamic and showed that the engaged topics were changed from the nature of COVID-19 to the vaccination and virus mutation which peaked in the early and middle terms of 2021. The public sentiment and engagement were shifted from hesitancy to anxiety towards the safety and effectiveness of the vaccines, whilst changed again into wariness on an uprising of the delta variant.

**Conclusion:**

Understanding public sentiment and opinion can help policymakers to plan the best strategy to cope with the pandemic. Positive sentiments and fact-based opinions on COVID-19, and COVID-19 vaccination had been shown predominantly. However, sufficient health literacy levels could yet be predicted and sought for further study.

## Introduction

Since first named as a global pandemic by the World Health Organization (WHO) in March 2020, COVID-19 has been the utmost issue challenging all aspects of human life worldwide. ^1^^,^^2^ Whilst, at the beginning of the crisis, little had been known about the pathogen, its detection, and its management; the disease began to increase the morbidity and mortality rate steeply and thus overwhelming the health care system in many countries, especially during the pre-vaccination period. ^3^

One of the keys to controlling the morbidity and mortality rate increase is vaccination allied with the implementation of WHO-recommended efforts for reducing transmission, together with strong epidemiology surveillance. ^4^ These are called for public literacy and engagement on COVID-19 and COVID-19 vaccination, which sentiments represent public opinion and beliefs toward the issues could be read on various platforms of social media including Twitter and might affect public acceptance toward the vaccine and vaccination program. ^3^^,^^4^ Although robust evidence has supported the efficacy and safety of various types of COVID-19 vaccines, public skepticism concerning vaccine effectiveness and side-effectd has become a significant shortcoming in achieving wide-vaccination coverage. ^5^

Anaylsis of social media content is a reliable way of mining for peoples opinions and beliefs, including toward COVID-19, COVID-19 vaccines, and vaccination; thus might help decision-makers to develop policies related to COVID-19 and COVID-19 vaccination. ^6^ The use of social media has been rising drastically and the analysis of public opinion uploaded on social media can be an effective means to capture real-time public sentiment. ^7^ Previous studies have been conducted on public opinion regarding COVID-19 vaccines and vaccinations in various countries; it was reported that the surge in COVID-19 positive cases was not necessarily associated with the levels of vaccinations. ^8^^,^^9^

In Indonesia, hesitancy towards the vaccine and low literacy levels affect the acceptance of COVID-19 vaccination or certain vaccine brands. ^10^ An early survey on vaccine acceptance from 1359 Indonesian respondents conducted from the end of March to April 2020, showed the acceptance rate as high as 95% for the free vaccine with reported efficacy of approximately 95%. However, the acceptance level dropped to 67% if the reported efficacy of COVID-19 vaccine was only 50%. ^11^ The Ministry of Health of the Republic of Indonesia stated that a survey on vaccination by the COVID-19 Symptom Survey conducted by the University of Maryland Joint Survey Methodology Program in partnership with Facebook showed several factors cause doubts about vaccination acceptance amongst Indonesian people e.g. concern about the side effects, and the comorbidity that may affect the post-vaccination health state. ^12^ Although Indonesia has the fourth-highest number of social network users worldwide, ^13^ studies on social media data to identify public sentiments and engagement towards COVID-19 and COVID-19 vaccination have still been limited, and thus is the aim of the current study.

## Method

### Ethics

This study received approval from the Health Research Ethics Committee (KEPK), Faculty of Medicine, Universitas Airlangga, Indonesia (approval no. 145/EC/KEPK/FKUA/2021).

**Figure 1.**
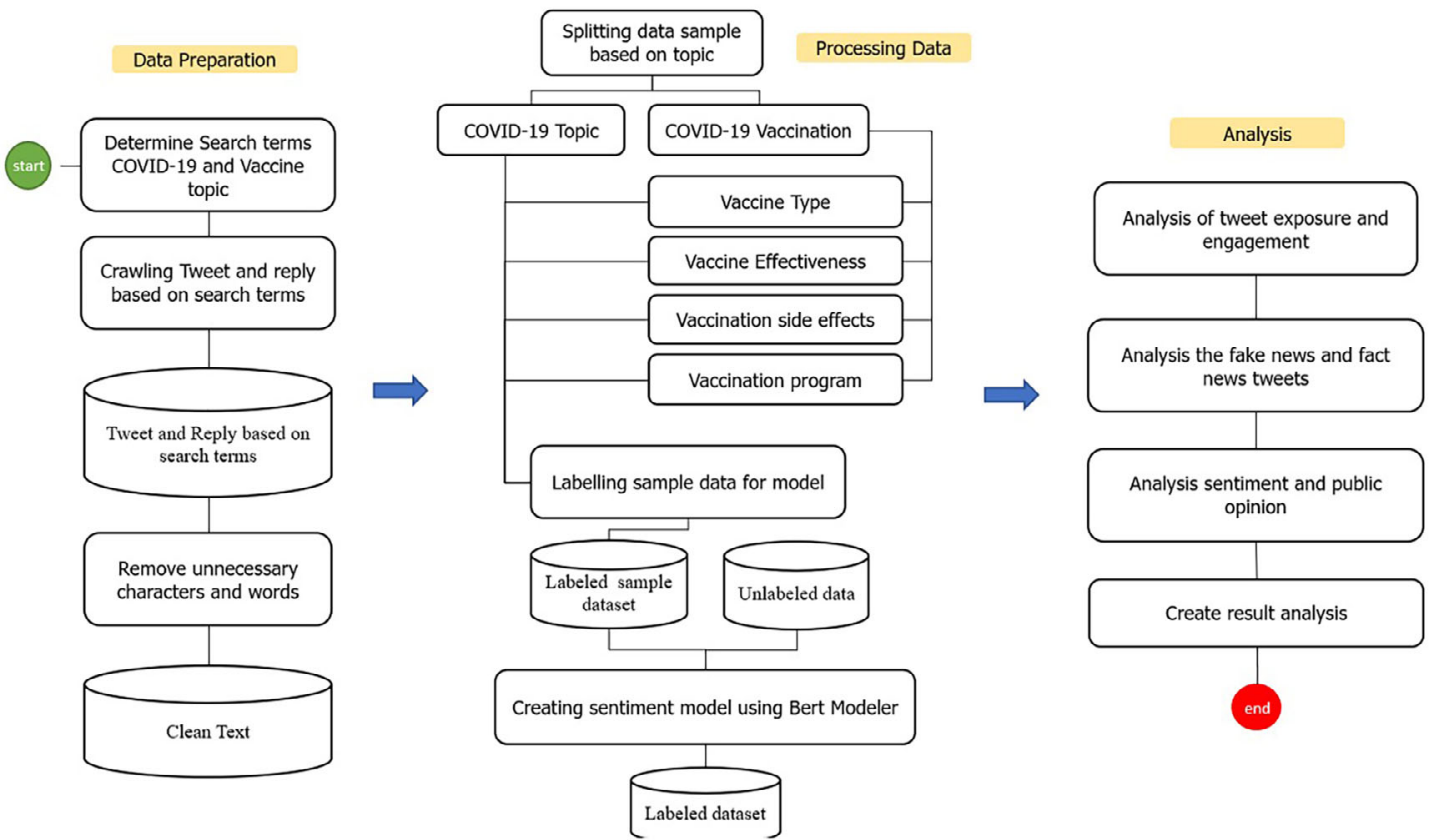
The process stages of the dataset on Indonesia Tweets during 1st of January, 2020–31st of August, 2021.

Several stages were carried out in this study to identify and analyze public opinion ( Figure 1). The preprocessing started with data preparation which included determining searching terms for COVID-19 and COVID-19 vaccinations, then crawling the Indonesian-language tweets data based on the meta-tagging language stored on Twitter ( https://twitter.com/?lang=en-id) during the period of 1 ^st^ January 2020 to 31 ^st^ August 2021.

The collected data were clustered into categories according to the search terms listed in Table 1. Subsequently, the data was cleaned from several elements such as emoticons, hashtags, non-alphanumeric characters, and URLs. The lower-text was used to convert all Twitter text to the lowercases. We then removed the punctuation in the Tweet text. The samples of dataset were taken from the cleaned data. The sample dataset was subjected to manual labeling within 3 categories, namely positive, neutral, and negative. We developed the sentiment model by using the manually labeled data and IndoBERT as tokenizer. The sentiment model was applied to run the labeling of all remaining data ( Figure 1).

**Table 1.** Searching terms COVID-19, COVID-19 vaccine and vaccination.

Category	Searching Terms
	COVID-19
	COVID-19, corona, corona virus, corona virus, SARS COV-2, COVID, masks, keep your distance, physical distancing, social distancing, washing hands, PPKM, PSBB, lock down, WFH, LFH, online learning, self-isolation, swab, PCR, 3M, 5M, 6M, tracing, comorbid COVID-19.
	COVID-19 Vaccine and Vaccination
Vaccine Type	COVID-19 vaccine, Sinovac, Chinese vaccine, Nusantara vaccine, red and white vaccine, biopharmaceutical vaccine, inactivated vaccine, mRNA vaccine, Zeneca Astra vaccine, Pfizer vaccine, Moderna vaccine.
Vaccine Effectiveness	Immunity, prevent COVID transmission, COVID positivity rate, positive COVID, herd immunity, death rate; delta variant, delta strain, variant of concern, prevent severe COVID, prevent ICU needs, prevent MRS needs.
Vaccine Side Effect	KIPI, post-immunization co-occurrence, paralysis, blood clots, blood clots, vaccine death, death after the vaccine, allergy, positive for COVID-19, drowsiness, hunger, sexual disorders, vaccine side effects, vaccine hazard, stroke, Guillain-Barre syndrome, pain, swelling, dizziness, headache, fever, muscle aches, vaccine chip.
Vaccination	National COVID vaccination, national COVID immunization, vaccination acceleration, mass vaccination, health worker vaccination, elderly vaccination, child and adolescent vaccination, third dose vaccination, booster, vaccination stage 1, vaccination stage 2, vaccination stage 3 vaccination, vaccination of BUMN, vaccination certificate, vaccination fake, self-vaccination, vaccination age 12-17, vaccination age 18-59, cooperation vaccination, paid vaccination, vaccination comorbid.

The data was subjected to exploratory data analysis (EDA), in order to to find out the insight about the data, to discover patterns, to spot anomalies, and to check assumptions with the help of statistics and graphical presentations. ^14^ The yield data was presented as the distribution of sentiment. The metric of tweet exposure was presented by word cloud and by the graph that depicted the dynamic changes by month within the captured period. The metric of tweet engagement involved the measurement of the metadata accompanying posts, including the number of retweets, likes, and replies. We also identified the circulating facts and the fake news on Twitter ( Figure 1). Python visualization was used for data presentation. ^15^

### Determination of the fact and fake news

The string comparison method was used to assess fact or fake tweets. This method compared tweets with the list of fake tweets on a dictionary issued by Turn Back Hoax. ^16^ The *difflib* library was used to obtain the similarity value between tweets. In determining fake news labeled tweets, it is necessary to have an additional parameter in the form of a threshold to tolerate the similarity between a tweet that can be considered a fake tweet and a tweet that is still considered factual. The range of similarity values was determined based on a sample test by taking into account the data results with a range of 0 – 1, where the closer the value to 1 means the more appropriate the word is in the list of incorrect tweets dictionary. After observing the tweet data, the threshold value was determined to be 0.7. The tweets with a similarity value above 0.7 would be categorized as fake tweets ( Table 2).

**Table 2.** Tweets that were indicated as the fake news showed invalid sources that could not be traced from the valid references (e.g. official release from the government, scientific articles of peer-reviewed journals).

Username	Tweet	Content classification
infocovid19_id	#HoaxBuster [SALAH] 21% Pasien Mengalami Efek Samping Setelah Memakai Vaksin Moderna Selengkapnya: https://t.co/Nh3DxlJIbr	fake news
cirtbuleleng	Dunia Setujui Vaksin Nusantara https://t.co/iWBlfFBTXM	fake news

### Analysis of public opinion and sentiment

Datasets from public and private accounts that had gone through the preprocessing stage were labeled as positive, negative, and neutral, as well as knowing the pattern of agreement on vaccination program data that have been marked as positive (pro-vaccination), negative (anti- and doubtful toward the vaccination) and Neutral which refers to the study. ^17^ Pro vaccines are categorized for tweets with a positive tendency towards vaccination. Tweets show that the public can well accept the existence of vaccines and invitations to participate in vaccinations, even when giving an opinion about their condition after the vaccination process, commonly known as adverse event following immunization (AEFI). Anti-Vaccine is given in tweets that reject vaccination, accompanied by arguments against it. Doubt was given to tweets that tend to be confused about the purpose of vaccination or still doubt the effectiveness of certain vaccine brands, such as wanting only Pfizer vaccines and disparaging other brands of vaccines. Neutral category tweets were usually dominated by news accounts and only inform facts or narratives without expressing an opinion that says whether the statement is negative or positive towards vaccination. ^5^^,^^6^^,^^11^

A total of 3000 data were taken randomly from the dataset to be used as training data using the IndoBert model, with an accuracy rate of 75%. IndoBERT constitutes a self-contained Deep Learning model designed for Natural Language Processing (NLP), inspired by the Transformer model. Each output element in this model is intricately connected to every input element, with dynamically computed weights based on inter-element relationships. BERT is formulated to aid computers in comprehending the ambiguous meaning of language within a text by utilizing the surrounding text to establish context. This model is trained using over 220 million words in the Indonesian language. In this study, IndoBERT is employed for the tokenization process of words before engaging in the classification task. Following the successful tokenization of the Indonesian language by IndoBERT, the data proceeded directly to the classifier layer to discern patterns in word tendencies, classifying them into three categories: positive, neutral, and negative. ^18^

## Results and discussion

A total of 3,489,367 Indonesian tweets data were collected from 1 ^st^ January 2020 to 31 ^st^ August 2021. The total dataset obtained was 324,358 data; consisting of 24,579 data related with COVID-19 and the rests were related with COVID-19 vaccine and vaccination.

**Figure 2.**
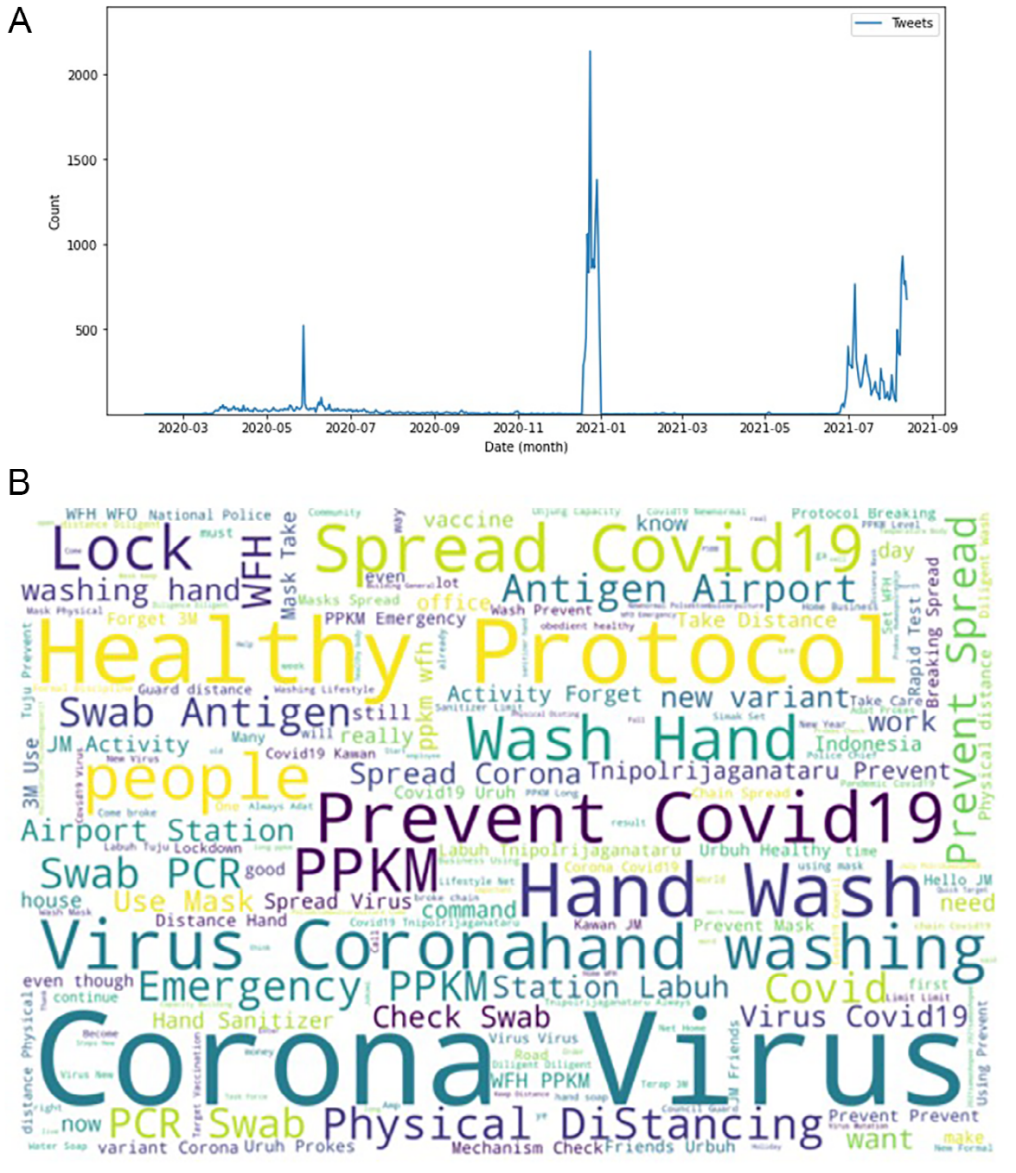
Tweets distribution on COVID-19 during 2020-2021. The peak counts were seen in June, 2020; in January, 2021; and in July, 2021 (graph). Word-cloud on COVID-19 were dominated by keywords of the virus, health protocols, detection methods and travelling protocols (words).

Figure 2 showed the peak counts of tweets were seen in three parts during this period of time. In June 2020; in January 2021 and again in July 2021. The word-cloud of these tweets were seen in this figure, e.g. corona virus, health protocol, COVID-19 spread, preventing COVID-19, PPKM (abbreviation of Indonesian government program to control COVID-19), hand washing and physical distancing. Several keywords i.e. PCR (polymerase-chain reaction), antigen swab and transportation protocols dominated the tweets.

**Figure 3.**
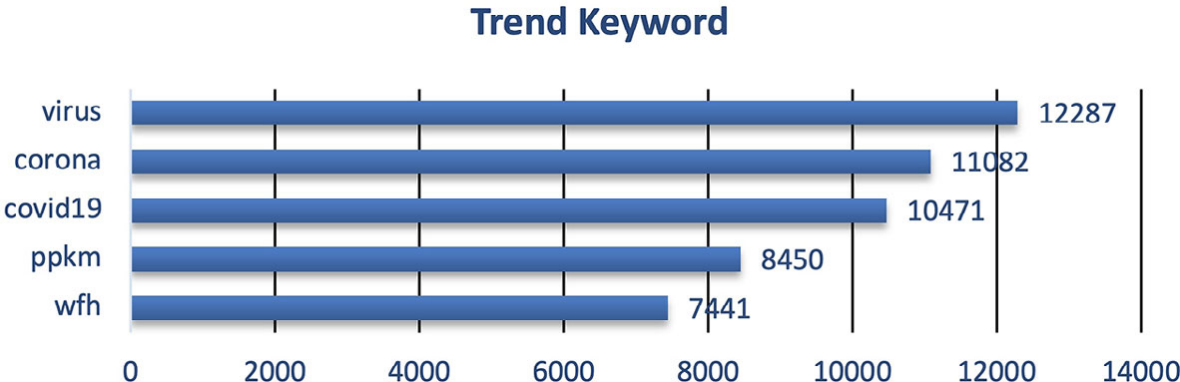
Trend keyword on the tweets showed that the public engagement and exposure during 2020 were predominantly on the virus, corona, COVID-19, PPKM and working from home/WFH, respectively.

During 2020 when the vaccine and vaccination programs had yet known, the trending keywords were more into the COVID-19 virus and how to take a preventive step i.e. PPKM (pemberlakuan pembatasan kegiatan masyarakat)/restrictions towards community activity), and working from home/wfh ( Figure 3). The word count graph shows many Twitter users discussed the COVID-19 virus along with tweets containing education to prevent exposure to the virus. During that period, the topic of discussion was still about education to suppress the spread of COVID-19. Preventive actions still being promoted, such as staying at home, avoiding direct contact with other people, avoiding non-essential travel, social distancing, frequent hand washing, and so on ^19^^,^^20^ remain hot topics among the public. Meanwhile, the word count shows a hot topic among the people after implementing PPKM. After the government established and implemented the PPKM policy, Twitter users discussed correlated issues such as working from home. ^21^^–^^23^ PPKM is intended as a form of response to the increase in COVID-19 cases, so the problem of the virus is still quite busy being discussed on Twitter. Implementing PPKM levels 1-4 by the government, which has brought pros and cons to the community, has also become a topic of discussion. Even so, this policy is considered effective in suppressing the surge in the increase in COVID-19 cases. ^21^

**Figure 4.**
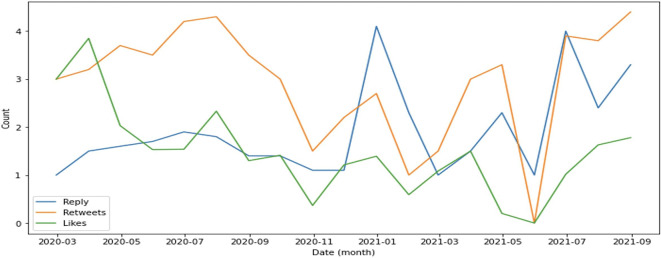
Engagement on COVID-19 were predominated by retweets during 2020-2021, except at the end of 2021 and in July, 2021 were the reply was higher than retweets and likes.

In general, retweets dominate engagement, followed by replies ( Figure 4). This supports evidence that the public is more focused on sharing information such as the rise or fall of cases as well as information on education and preventive measures implemented by the government to suppress the spread of COVID-19. ^22^

On the other hand, the tweets count on COVID-19 and COVID-19 vaccination was highest at July 2021 ( Table 3), arguably due to the rise of SARS-CoV-2 delta variant that significantly increased the morbidity and mortality rate, thus called for more definitive prevention act. ^22^^–^^24^

**Table 3.** Tweets count on COVID-19 vaccine and vaccination during 2020-2021, it was shown that the highest was in July, 2021; followed by those from the start of 2021 until March, 2021.

Month	Count (Tweets)	
	2020	2021
January	1	1392
February	0	1066
March	2	1837
April	4	558
May	1	830
June	4	1347
July	13	13599
Agustus	73	7015
September	101	N/A
October	186	N/A
November	216	N/A
December	1682	N/A

**Figure 5.**
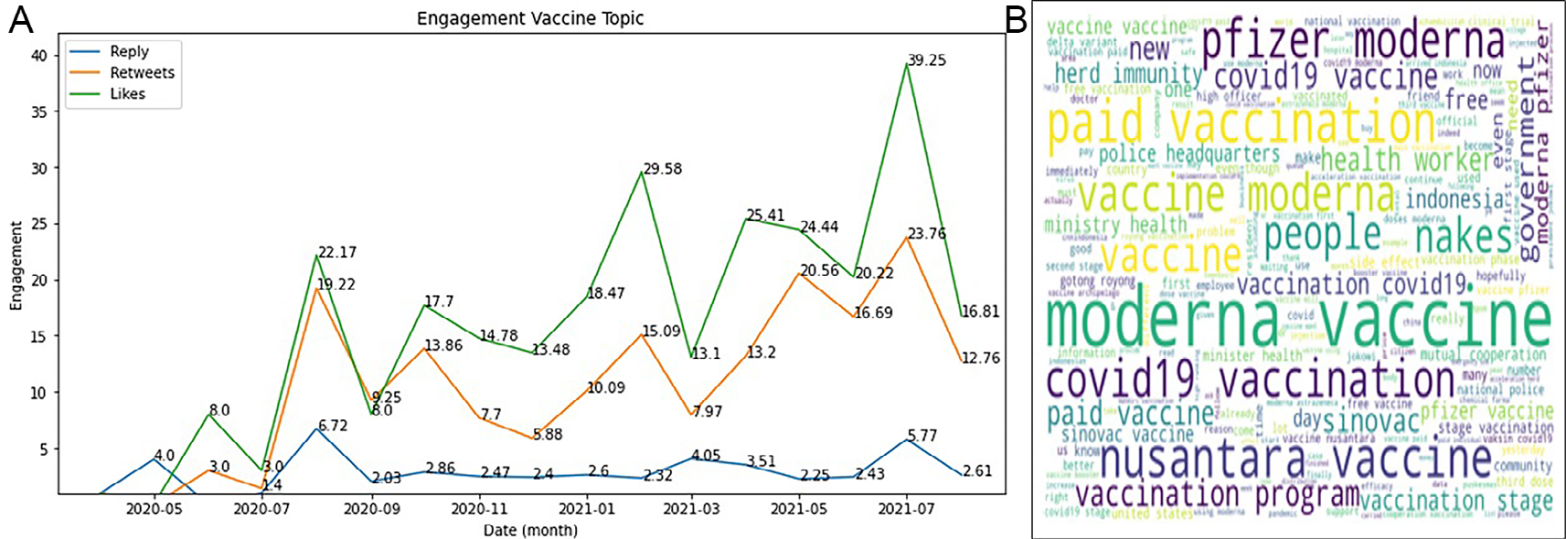
Engagement on the vaccine of COVID-19 during 2020-2021 was shown to be highest at July, 2021 by the likes, followed by retweets and reply, respectively (graph). The word-cloud of this topic was predominated by the kind of vaccine brands (words).

The engagement on COVID-19 vaccine was topped by the likes during 2020-2021, followed by retweets and reply ( Figure 5). In Indonesia, several brands that have gone through safe and halal tests that adapt to the conditions of Indonesia, being a country with one of the largest Muslim population in the world, are exciting topics to be discussed by the public. ^23^ For example, the most popular brand in Indonesia is Sinovac. ^24^ This is because Sinovac is the fastest vaccine brand to enter Indonesia. ^25^ It can be seen from the data that has been successfully presented that Sinovac continues to dominate, especially at the end of 2020 and early 2021, when the Sinovac vaccine has entered Indonesia. The Moderna vaccine also experienced an increase in the number of tweets because, during this period, many of these vaccines were distributed in Indonesia. ^26^ Moderna vaccine produces more side effects than other vaccines, ^27^ thus becoming increasingly discussed on Twitter. The AstraZeneca vaccine also carries more frequent side effects when compared to the Sinovac vaccine ( Figure 5). ^28^

**Figure 6.**
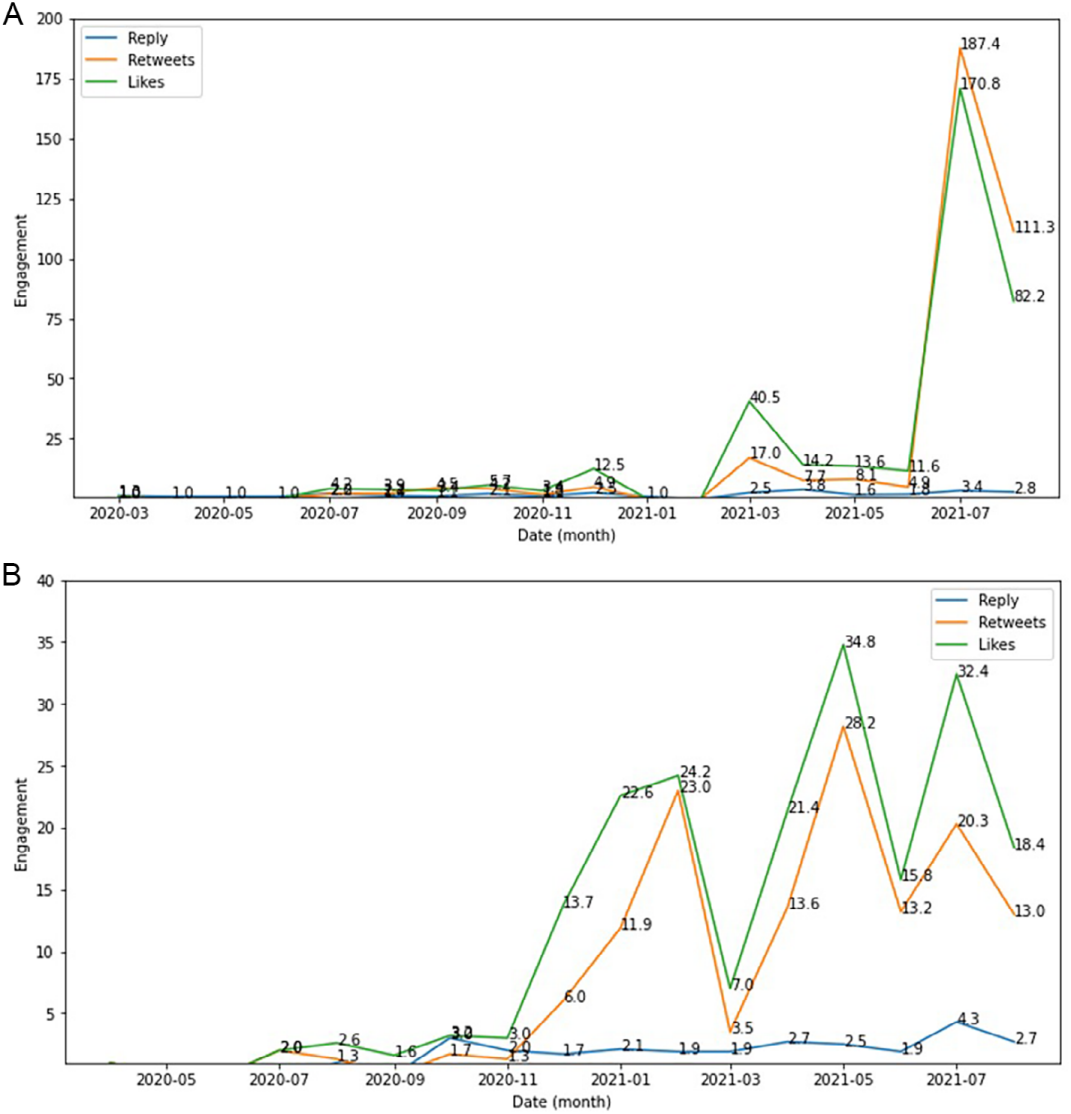
Engagement ratio on the side effect of COVID-19 vaccine was shown highest at June-July, 2021; followed by in March, 2021 and at the change of the year from 2020 to 2021 (top). The below chart showed the engagement level of vaccine type in this period, which topped by the likes and followed by retweets and reply, respectively.

The side effect of COVID-19 vaccine was seen as the engagement topic topped by likes, retweets and reply which were higher in December, 2020; in March, 2021 and in July, 2021 compared to other months during this period. Whereas the type of vaccine engagement showed higher in February 2021; in May 2021 and in July 2021 compared to other months ( Figure 6).

**Figure 7.**
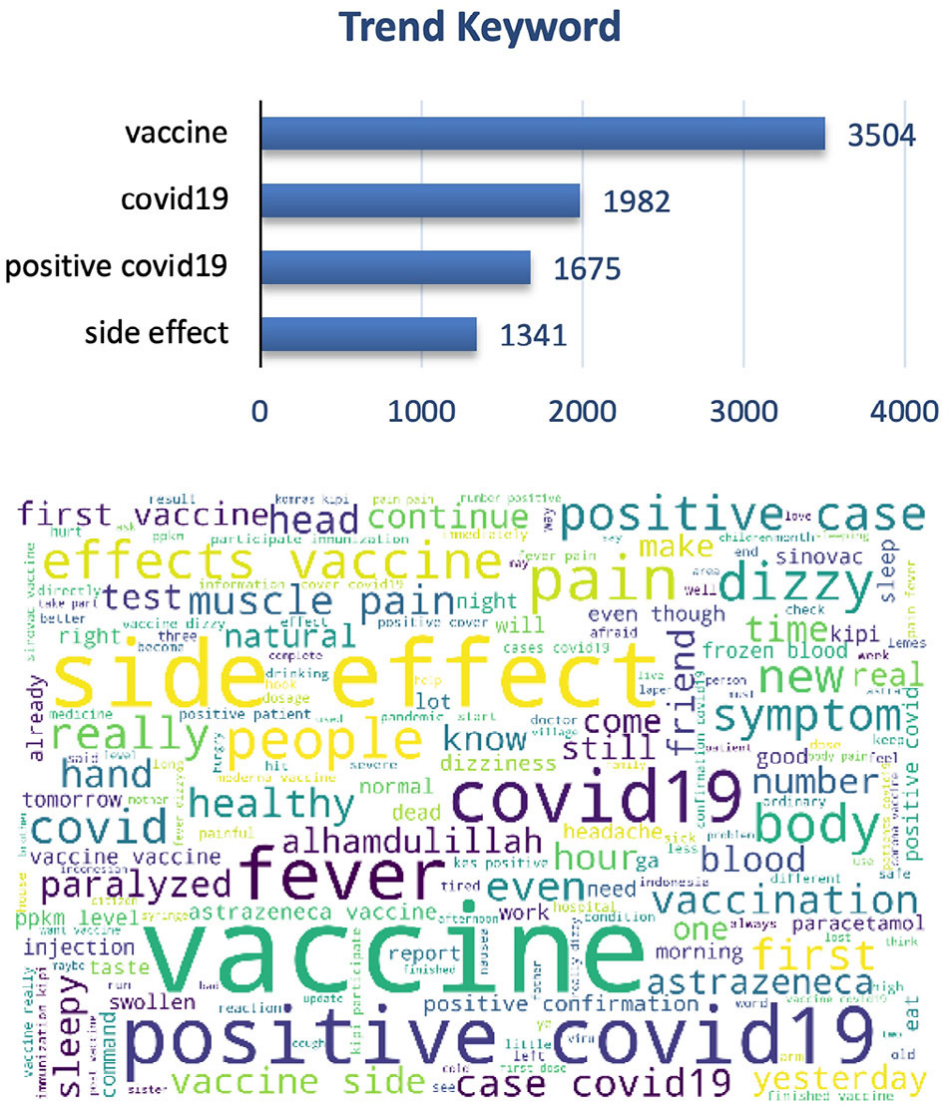
Word-cloud of vaccine side effects subtopics, predominated with the trend keywords e.g. Vaccine, COVID-19, positive COVID-19 and side effect.

The graphs and word cloud show that of the vaccine side effects subtopics recorded, the word most frequently discussed in tweets was COVID-19 vaccine and its side effect ( Figure 7).

**Figure 8.**
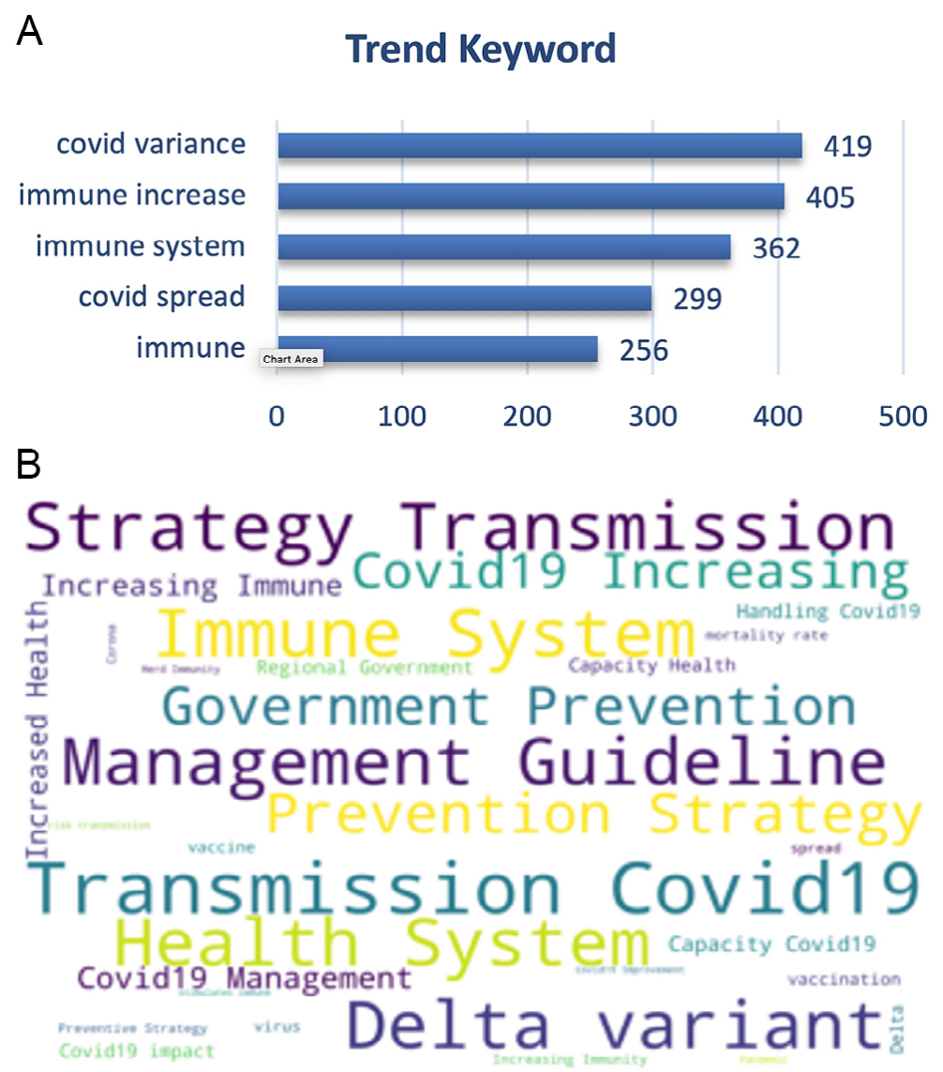
Word-cloud of the vaccine effectiveness subtopic based on the searching terms were dominated by the transmission of COVID-19, delta variant, strategy of prevention, management of the disease and preventive strategy.

On the other hand, the trending keywords on the vaccine effectiveness was topped by trending keywords of the disease transmission, management guidelines, virus variance e.g. delta variant also with the immune system, prevention and health system ( Figure 8).

**Figure 9.**
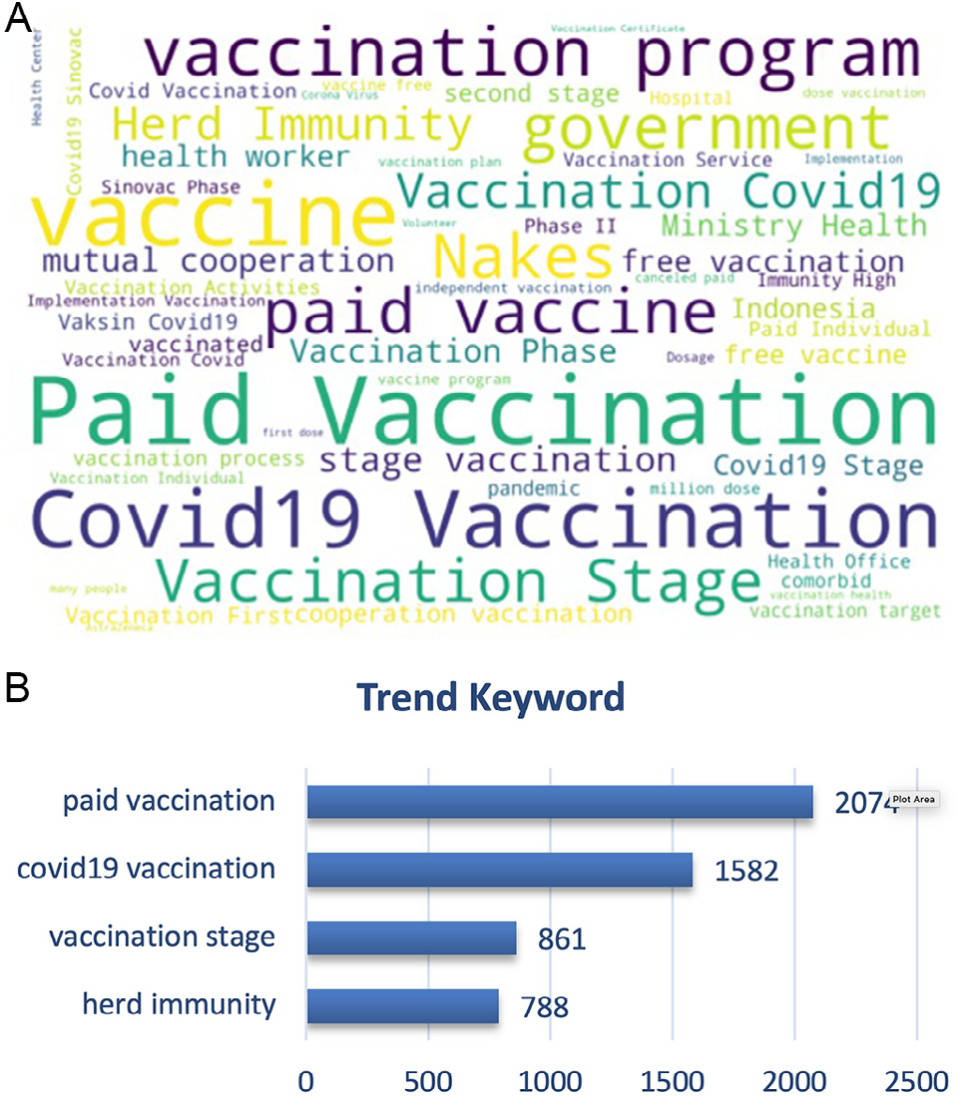
Word-cloud of the vaccination sub-topic, where paid vaccination, vaccination stage, government vaccination program and free vaccination became the trending keywords.

From Figure 9, we can see that the paid vs. free vaccination programs became the trending keywords along with the vaccination program of the government and herd immunity. People were cautious on the paid vaccination, when in reality the vaccination program was held nationally since February 2021 by the government with primary target of healthy people aged 18-59 years old, and also prioritized for the health providers to instigate the herd immunity that was discussed as the ideal condition after vaccination coverage was achieved widely. ^22^^–^^24^

Based on the analysis using similarity value, 15 tweets were indicated as fake tweets with misleading content. Some of them include the worldwide approval of a dendritic cell-based vaccine candidate developed by a group of Indonesian researchers, which in fact had not even been authorized into phase 2 clinical trial by the Indonesian authoritative body. There was also content about the affiliation of several vaccine manufacturers to certain companies. Another topic was that up to 21% of vaccine trial participants experienced adverse effects after receiving the Moderna vaccine. The fake tweets of vaccine manufacturing companies presumed to have been developing COVID vaccines even before the COVID-19 pandemic started was also found to be misleading content. From the entire dataset, it can be concluded that there was extremely low tweet activity of spreading fake news with a negative context, such as finding supporters for fake news from tweets that are replied.

**Figure 10.**
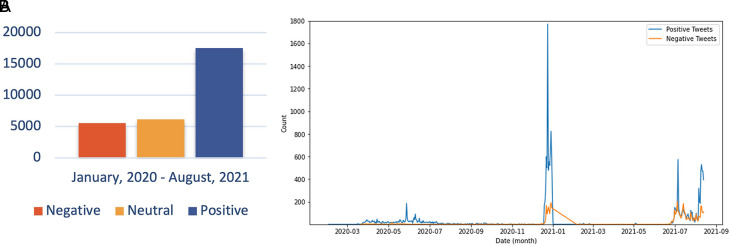
Public Sentiment about COVID-19 was predominated by the positive opinions, whilst the neutral and negative sentiments were followed, respectively by the approximately half of the prior sentiment (graph). The chart showed that the positive sentiment outweighed the negative sentiments and were peaked in June, 2020; in January, 2021 and in July, 2021.

The sentiment referred to in the discussion includes users agreeing and understanding the conditions for COVID-19. The debate regarding the support for preventive actions by the government is also a topic that is still hotly discussed. In addition, Twitter users continue to carry out their activities as usual and provide education on how to prevent exposure to the virus, increasing positive sentiment about the issue. In the second period, the trend of positive sentiment led to discussions around the expressions of Twitter users to express their response to the increasing number of COVID-19 cases in Indonesia, accompanied by campaigns from all parties to carry out vaccinations aggressively. However, there was an increase in negative sentiment in the second period compared to the first period. This period was when the government begins to issue PPKM policies that reap the pros and cons of the community. PPKM, which has a level of 1-4, was arguably considered to harm the community’s economy because of the limited activities of the community at work. During PPKM 3-4 part of rules was closing the purchasing center at 20.00 GMT+7, and making the visitor capacity a maximum of 50%. ^29^ Thus, many parties have complained about the condition of the policy. The sentiment distribution every monthis presented as shown in Figure 10.

**Figure 11.**
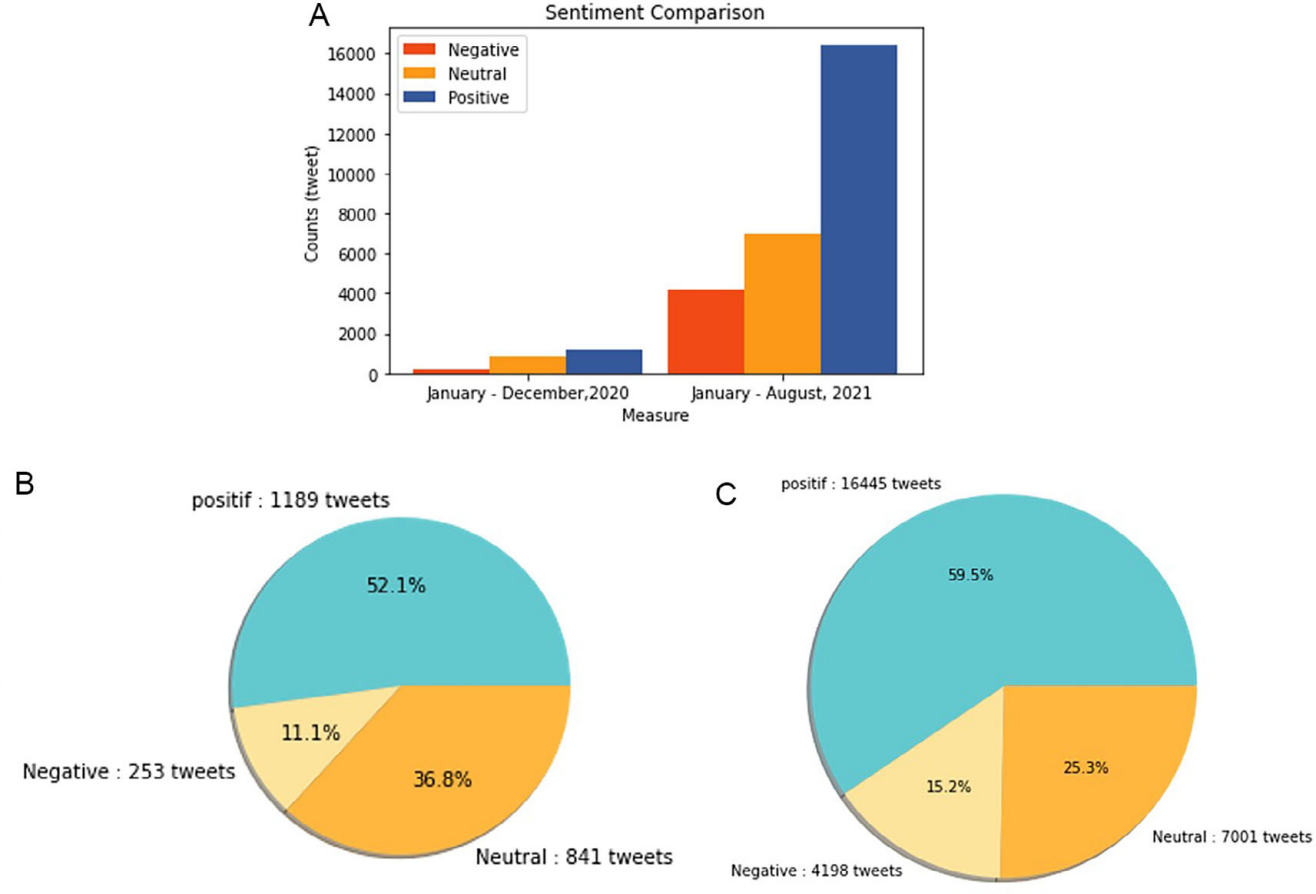
(A) Vaccination sentiment analysis comparison between 2020 and 2021 (graph). The sentiment of COVID-19 vaccination program in 2020 (B) and 2021 (C) showed in the pie charts, respectively, where in both period the positive sentiment outweighed the negative sentiment by approximately 5 times.

Sentiment analysis was conducted to find out the positive and negative sentiments of the public towards the vaccination program in 2020, shows that positive tweet sentiment dominates all existing sentiments. Analysis of the dataset indicates that generally, tweets come from news accounts where tweets originating from these accounts are classified as neutral tweets. While the period of 2021, shows that although the number of tweets is more dominant than in the first period, it shows that public opinion has a positive sentiment tendency. This might be due to the public rise of awareness of the importance of vaccination to help reducing the spread of the COVID-19 virus. However, negative sentiment is still a significant problem because people expect only certain types of vaccines would work, and/or are still doubtful and do not believe in vaccination programs to tackle the pandemic ( Figure 11).

**Figure 12.**
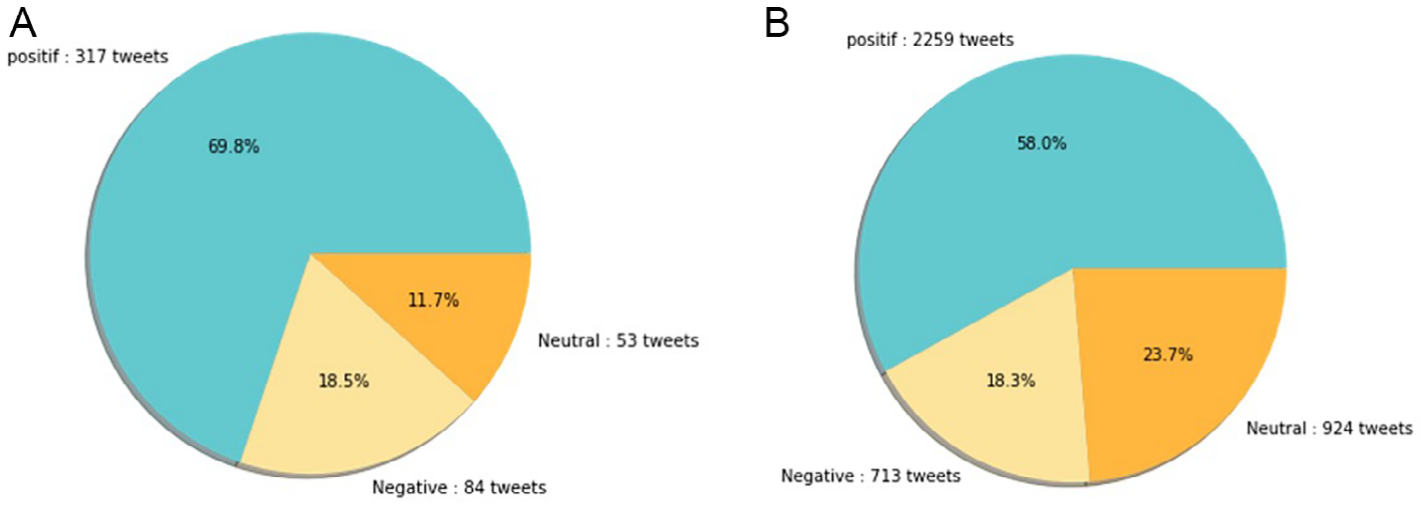
The sentiment on the side effect of COVID-19 vaccine in 2020 and 2021 showed by the left and the right pie charts, respectively. The positive sentiment was higher than the negative or the neutral sentiments in both years.

Figure 12 shows that both in 2020 and 2021, positive sentiment tends to dominate above 50%. The proportion of positive sentiment decreases considerably while negative sentiment only slightly decreases in 2021. The percentage of neutral sentiment doubles in 2021 compared to 2020. Negative sentiment can happen because, in 2020, the existing vaccine research is still in the development stage by scientists, while during the first midterm of 2021, COVID-19 vaccines are still being rolled out to limited people in Indonesia.

In 2021, various vaccine products have gone through stages of trials and study results of their efficacies and side effects have been released to public by vaccine manufacturers. Due to the increasing clarity of reports regarding the effect of vaccine in stimulating the body’s immunity against virus, the public has more confidence in the role of vaccines in accelerating recovery from the pandemic. It is reflected by slightly increased positive sentiment of vaccination program in 2021 ( Figure 11). However, fear toward vaccine’s side effect yet exists, possibly contribute to the decrease of positive sentiment in 2021 ( Figure 12).

**Figure 13.**
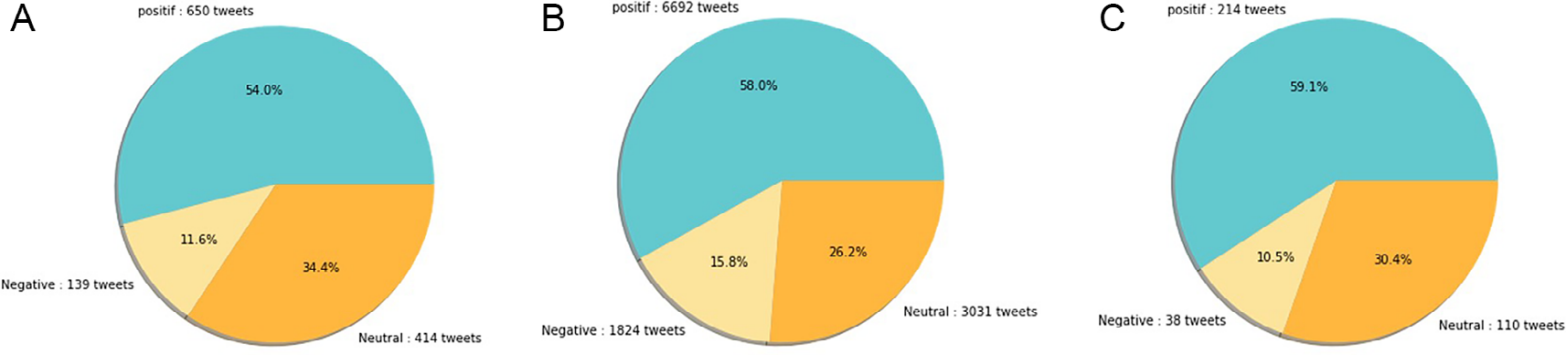
The sentiment on COVID-19 various type of vaccines in 2020 (A) and 2021 (B) showed that the positive sentiments outweighed (C) the negative and neutral sentiments; the pie chart showed the public sentiment on the effectiveness of the vaccines in government vaccination program that was predominated by, again, positive sentiment followed by the neutral and negative sentiments.

At Figure 13, the sentiment of the vaccination program in Indonesia was mostly positive (59.1%); whilst the analysis sentiment of various types of vaccines for COVID-19 that were available was predominated by positive opinions in general, during both years (approximately five times higher than the negative sentiments). We compared sentiment polarities toward vaccines in 2020 and 2021 and found increases in both positive and negative sentiments in 2021 after the vaccination program had started. Although positive sentiments showed a greater increase, the negative sentiments, which remained at 15.2%, deserve attention, as they may reflect a proportion of individuals with vaccine hesitancy and/or rejection.

Social media studies have been used over the past decade to identify public opinion and sentiment toward particular health issues, for instance, the 2009 H1N1 outbreak. ^30^ The vaccination issue has been a matter of importance to be analyzed using this approach since the individual decision on vaccine uptake is modulated by opinions from social networks. ^31^ Disinformation narratives spread on social media that are sometimes hostile, causing anxiety, fear, and distrust toward vaccination could largely contribute to vaccine hesitancy and refusal. ^32^ The rapid and vast dissemination of disinformation should be addressed appropriately by effective strategies for vaccine promotion. The surveillance of real-time social media information flow could be a remarkable source of timely data updates for adjusting those strategies. ^31^ Interestingly, a strong correlation was shown between online-expressed sentiments and estimated vaccination rates. ^33^

Recent studies have confirmed evidence of vaccination impacts on several public health parameters, which give promise for its role in achieving herd immunity and further ending pandemic. Vaccination has remarkably reduced COVID-19 cases and hospitalizations. ^34^ COVID-19 related morbidity and mortality, ^35^ and incidence due to variants of concern. ^36^ Additionally, rapid vaccine roll-out is believed to boost economic recovery. ^37^ Therefore, accelerating the vaccination pace is imperative for countries worldwide. ^34^ Efforts to lower vaccine hesitancy should be prioritized as it is the greatest threat to achieving high vaccine coverage. ^38^ Social media is perceived to be a major source of misinformation and is able to amplify and disseminate it without temporal and spatial limits, fostering vaccine hesitancy and lowering vaccine uptake. ^39^ Our study identified a number of vaccine-related misinformation circulating on social media that were categorized as fake tweets. Our finding is in accordance with a study by Islam *et al*., that analyzed rumors and conspiracy theories related to COVID-19 vaccine on variable online platforms. They encountered that the majority of these contents were false and/or misleading. They also reported that Indonesia was among the countries with a high number of online rumors related to COVID-19 vaccine. ^40^ Our study found 15 vaccine-related fake tweets. Even though they were quite low in number considering the dataset collected was within 20 months, their impact on vaccine hesitancy shouldn’t be undermined. ^41^ We found fake tweets about the adverse effect of Moderna vaccine that was said to affect 21% of trial participants. This exaggerated misinformation certainly may raise public concern about vaccine safety, which may lead to vaccine hesitancy. ^42^ Another fake tweets issue was that vaccine manufacturers had been developing COVID-19 vaccines long before the pandemic emerged. This issue appeared to arise due to doubt about the fast pace of vaccine development. Some individuals may furtherly link it to the conspiracy theory that COVID-19 is a bioweapon designed by particular countries or parties. Meanwhile, belief in conspiracy theories is a driving factor for an individual to reject vaccination. ^43^^,^^44^ Actually, Twitter has set policies and conducted efforts to counteract any types of misinformation. It has launched detailed criteria and examples of false or misleading information about COVID-19 vaccine posted by users. Some actions taken to violations include content removal, tweet labeling, and adding corrective information. Twitter may also disable retweets, quoting, or any other ways of engagement to those false tweets, in case they pose potential harm to the public. The user’s account may be temporarily locked or permanently suspended. ^45^ It is very likely that Twitter has effectively cleansed any circulating misinformation. In addition, the ministry of health and the ministry of communication and informatics have provided platforms to continuously inform the public about the identified fake news or hoax and cooperate with social media to monitor misinformation related-contents and provide authoritative information as valuable trusted sources. ^46^ However, social media remains a battleground where the anti-vaccine movement is difficult to combat. ^47^

Despite the limitation that the collected opinion of Indonesian Twitter users might not fully reflect those of whole Indonesian population and that the used search terms might potentially cause overlap between the “COVID-19” and the “COVID-19 Vaccine and Vaccination” subsets, this study successfully confirmed the usefulness of social media studies to provide insights into the public’s attention, discussion, concerns, and sentiments about COVID-19 and COVID-19 vaccines and vaccination. ^48^ We demonstrated the dynamically changing public attention over time, where it peaked in July 2021, during the second surge of COVID-19 cases. It can be extrapolated that the public well responded to the government campaign of accelerating vaccine rollout as a means to curb the disease. ^49^ The top trend discussion topics may reflect a public concern, for instance, “paid vaccination. Even though we did not perform sentiment analysis on this specific topic, it can be proposed that the public highly disagreed with this policy.

We deep-dived user-generated Twitter posts to elaborate on factors associated with vaccine hesitancy. Other than the spread of misinformation through social media, we discovered the public’s perceptions and concerns about vaccines effectiveness and side effects. The negative sentiment toward these topics may be associated with the public’s doubt regarding the vaccine as an effective and secure means to manage the pandemic. Our result was in line with a study on medical students reporting most of the participants had concerns regarding vaccines adverse effects and ineffectiveness. ^50^ We identified a potential misconception regarding the side effects of the vaccine that it may indeed cause COVID-19 infection. This was actually one of the false myths identified and clarified by health authorities, yet still remains to be popular according to our findings. In this study, we found that people’s opinion showed on this particular social media is arguably correlated with their literacy on the issue. ^51^ It might be affected by the ambience of social environment and information percepted at a certain period.

## Conclusions

The public opinion and sentiment analysis on social media using an artificial intelligence of NLP may shortly provide timely data reflecting real-world public opinion. Thus, it should be part of the basis for developing strategies for public health response, particularly in a critical period of disease outbreak.

## Data Availability

The underlying data to this research cannot be shared due to the ethical and copyright restrictions surrounding social media data. The Methods section contains detailed information to allow replication of the study. Any queries about the methodology should be directed to the corresponding author.
