# Supplementary material for: Sentiment analysis of Indonesian tweets on COVID-19 and COVID-19 vaccinations
Source: F1000Res. 2024 Apr 16;12:1007. Originally published 2023 Aug 21. [Version 4] doi: 10.12688/f1000research.130610.4 (PMC11007366; doi:10.12688/f1000research.130610.4)
Supplement: Peer review history

# Reviewer report: Reviewer response for version 4

**DOI:** 10.5256/f1000research.164939.r266874

**Published:** 2024-04-17

**Authors:** Giuseppe Porro

**Affiliations:** 1 University of Insubria, Varese, Lombardy, Italy

No further comments from my end

Is the work clearly and accurately presented and does it cite the current literature?

Partly

If applicable, is the statistical analysis and its interpretation appropriate?

Yes

Are all the source data underlying the results available to ensure full reproducibility?

No

Is the study design appropriate and is the work technically sound?

Yes

Are the conclusions drawn adequately supported by the results?

Yes

Are sufficient details of methods and analysis provided to allow replication by others?

Partly

Reviewer Expertise:

sentiment analysis; subjective well-being evaluation; statistical methods for causal inference

I confirm that I have read this submission and believe that I have an appropriate level of expertise to confirm that it is of an acceptable scientific standard.

# Reviewer report: Reviewer response for version 3

**DOI:** 10.5256/f1000research.163487.r256878

**Published:** 2024-04-10

**Authors:** Aasif Ahmad Mir

**Affiliations:** 1 Pondicherry University, Puducherry, Puducherry, India

Paper analyses twitter communication about Sentiments of Indonesian tweets on COVID-19 and COVID-19 vaccinations

 gain some insights into overall public communication about the topic. It also shows a degree of positive, negative, and Neutral tweets gaining insights into dominant public opinion. Overall, it is a worthwhile contribution to the field. I would recommend the paper with some minor modifications.

**Comments to the Author**

**Abstract**

- Abstract is comprehensive and effectively summarizes the key points of the study.

**Introduction**

- Introduction is well written and provides detailed background of the study.

**Previous studies**

- The section describes the previous literature is missing.
-  Some important related studies are missing e.g. refer 1 and 2.

**Methodology**

 The article contains detailed methodology and presents everything clearly.

**Findings and Discussion**

- Findings are clearly explained and presented.

Is the work clearly and accurately presented and does it cite the current literature?

Partly

If applicable, is the statistical analysis and its interpretation appropriate?

Yes

Are all the source data underlying the results available to ensure full reproducibility?

Yes

Is the study design appropriate and is the work technically sound?

Yes

Are the conclusions drawn adequately supported by the results?

Yes

Are sufficient details of methods and analysis provided to allow replication by others?

Yes

Reviewer Expertise:

Social media analytics

I confirm that I have read this submission and believe that I have an appropriate level of expertise to confirm that it is of an acceptable scientific standard, however I have significant reservations, as outlined above.

## References

[1] : Public perception of COVID-19 vaccines from the digital footprints left on Twitter: analyzingpositive ,neutral andnegative sentiments of Twitterati. *Library Hi Tech* .2022;40(2) : 10.1108/LHT-08-2021-0261 340-356 10.1108/LHT-08-2021-0261

[2] : Sentiment analysis of Indian Tweets about Covid-19 vaccines. *Journal of Information Science* .2022; 10.1177/01655515221118049 10.1177/01655515221118049

# Reviewer report: Reviewer response for version 3

**DOI:** 10.5256/f1000research.163487.r255020

**Published:** 2024-03-18

**Authors:** Giuseppe Porro

**Affiliations:** 1 University of Insubria, Varese, Lombardy, Italy

Approved

Is the work clearly and accurately presented and does it cite the current literature?

Partly

If applicable, is the statistical analysis and its interpretation appropriate?

Yes

Are all the source data underlying the results available to ensure full reproducibility?

No

Is the study design appropriate and is the work technically sound?

Yes

Are the conclusions drawn adequately supported by the results?

Yes

Are sufficient details of methods and analysis provided to allow replication by others?

Partly

Reviewer Expertise:

sentiment analysis; subjective well-being evaluation; statistical methods for causal inference

I confirm that I have read this submission and believe that I have an appropriate level of expertise to confirm that it is of an acceptable scientific standard.

# Reviewer report: Reviewer response for version 2

**DOI:** 10.5256/f1000research.159111.r224744

**Published:** 2023-12-13

**Authors:** Giuseppe Porro

**Affiliations:** 1 University of Insubria, Varese, Lombardy, Italy

The issues raised have been properly addressed by the Authors.

 I have no further comments.

Is the work clearly and accurately presented and does it cite the current literature?

Partly

If applicable, is the statistical analysis and its interpretation appropriate?

Yes

Are all the source data underlying the results available to ensure full reproducibility?

No

Is the study design appropriate and is the work technically sound?

Yes

Are the conclusions drawn adequately supported by the results?

Yes

Are sufficient details of methods and analysis provided to allow replication by others?

Partly

Reviewer Expertise:

sentiment analysis; subjective well-being evaluation; statistical methods for causal inference

I confirm that I have read this submission and believe that I have an appropriate level of expertise to confirm that it is of an acceptable scientific standard.

# Reviewer report: Reviewer response for version 1

**DOI:** 10.5256/f1000research.143380.r206421

**Published:** 2024-02-27

**Authors:** Annisa Ristya Rahmanti

**Affiliations:** 1 Graduate Institute of Biomedical Informatics, Taipei Medical University, Taipei, Taiwan; 2 Department of Health Policy Management, Faculty of Medicine, Public Health and Nursing, Universitas Gadjah Mada, Yogyakarta, Special Region of Yogyakarta, Indonesia

- The work presents a clear and structured overview of the study, which focuses on analyzing sentiments regarding COVID-19 and vaccinations expressed in Indonesian-language tweets. However, while the author briefly mentions previous studies, the current research would benefit from a more comprehensive literature review. This would better contextualize the research within the broader scope of existing findings, particularly those focusing on studies conducted in similar contexts or regions. 
- The study design employs relevant tools such as the IndoBERT model for clustering and the difflib library for string comparison. However, the technical soundness is difficult to fully assess without more detail on the validation of these methods and their appropriateness for the analysis of Indonesian-language tweets. Could you provide the performance model?
- Although the results were presented using pie charts to illustrate sentiment trends, a time series analysis would provide a more accurate representation of how sentiments have evolved over time. Time series analysis can capture the dynamic nature of public opinion affected by fake news or government actions, allowing for a clearer understanding of trends and patterns that may not be immediately apparent in static visual representations such as pie charts.
- It would be pertinent to adjust the conclusion to acknowledge the methodological limitations and suggest more robust analytical methods, like time series analysis, for future studies. Additionally, addressing the ethical considerations involved in using social media data is also crucial.

Is the work clearly and accurately presented and does it cite the current literature?

Yes

If applicable, is the statistical analysis and its interpretation appropriate?

Partly

Are all the source data underlying the results available to ensure full reproducibility?

Partly

Is the study design appropriate and is the work technically sound?

Partly

Are the conclusions drawn adequately supported by the results?

Partly

Are sufficient details of methods and analysis provided to allow replication by others?

Yes

Reviewer Expertise:

health informatics, NLP, social media data analytics, AI

I confirm that I have read this submission and believe that I have an appropriate level of expertise to confirm that it is of an acceptable scientific standard, however I have significant reservations, as outlined above.

# Response

**Published:** 2024-03-07

**Authors:** Viskasari Kalanjati

**Affiliations:** Airlangga University, Indonesia

- Comment and suggestion: The work presents a clear and structured overview of the study, which focuses on analyzing sentiments regarding COVID-19 and vaccinations expressed in Indonesian-language tweets. However, while the author briefly mentions previous studies, the current research would benefit from a more comprehensive literature review. This would better contextualize the research within the broader scope of existing findings, particularly those focusing on studies conducted in similar contexts or regions. 
- **Answer: **Thank you for the insightful comment. We have added an explanation of our result in comparison with other studies in Indonesia. Please find it in the result and discussion chapter.
- The study design employs relevant tools such as the IndoBERT model for clustering and the difflib library for string comparison. However, the technical soundness is difficult to fully assess without more detail on the validation of these methods and their appropriateness for the analysis of Indonesian-language tweets. Could you provide the performance model?
- **Answer:** Thank you for the suggestion. We have added an explanation about the validation of IndoBert Model, and cited previous studies that have shown good performance and validity of IndoBERT model. Difflib is an established method of string comparison that is often used to examine the similarity of text. Please find further explanation at https://docs.python.org/3/library/difflib.html . 
- Although the results were presented using pie charts to illustrate sentiment trends, a time series analysis would provide a more accurate representation of how sentiments have evolved over time. Time series analysis can capture the dynamic nature of public opinion affected by fake news or government actions, allowing for a clearer understanding of trends and patterns that may not be immediately apparent in static visual representations such as pie charts.
- **Answer: **Thank you for the suggestion. We highly appreciate it. We recognize it as one of the limitations of our study and put it in a suggestion for future studies. 
- It would be pertinent to adjust the conclusion to acknowledge the methodological limitations and suggest more robust analytical methods, like time series analysis, for future studies. Additionally, addressing the ethical considerations involved in using social media data is also crucial.
- **Answer:** Thank you for the suggestion. We have made revisions accordingly. Please find it in the paragraph about the limitations of our study and also in the conclusion. 

# Reviewer report: Reviewer response for version 1

**DOI:** 10.5256/f1000research.143380.r219774

**Published:** 2023-11-15

**Authors:** Giuseppe Porro

**Affiliations:** 1 University of Insubria, Varese, Lombardy, Italy

The paper offers a sentiment and opinion analysis about the reactions to Covid-19 pandemic, vaccination strategies and measures of social restrictions in Indonesia. The data source is a collection of Twitter messages posted between January 2020 and August 2021.

 The topic is interesting, its relevance is well argued and the results are properly presented and discussed.

 My main remarks concern the opportunity of providing more detailed information about the methodology the Authors apply in the study. 

 The unavailability of the data (due to privacy constraints) makes it necessary to be clear about the analytical procedures illustrated in Fig.1, in order to ensure the replicability of the study, albeit with different data and to avoid, as far as possible, any "black box" effect.

 In particular:

 a) I assume that the data were labeled into the categories indicated in Table 1 in the downloading stage. Then they were further classified into "classes, which were tweet exposure and engagement analysis" (p.3): how was this classification stage performed?

 b) before classifying data, a cluster analysis was applied using IndoBERT model. What is the role of cluster analysis in the study? Is there any relationship between cluster analysis and classification stages? Or, alternatively, is cluster analysis only aimed at visual inspection of data (in this case, it should be described before the classification stage, in order to avoid misunderstanding)? 

 c) please, provide details on how the sentiment analysis is performed: how is a tweet evaluated as positive, negative or neutral? At p.5 (point 2) the manual classification of a 3000-texts-size random sample is described and indicated as a training set for the IndoBERT model. How does the IndoBERT model work? Is the 75% accuracy rate the result of a test by the Authors themselves?

 d) p.2, point 2: the description of the content of positive, negative and neutral tweets is accompanied by a sort of opinion analysis: is it the result of a reading of the training sample or has an opinion analysis been actually extended to the whole dataset?

 e) despite the low incidence of fake tweets, the Authors invite not to undervalue the impact of fake news. Is there any way for identifying a fake news diffusion pattern, following retweets, replies and "like"s, as these seem to be available pieces of information (see p.6)?

 In order not to move the focus of the paper from a public health to an artificial intelligence one, going into a more detailed description of the methodology may require a methodological appendix, where some examples could also be provided.

 Minor remarks:

 - as usual, when a sentiment or opinion analysis is performed via social network sites data, some words should be spent to comment the lack of representativeness of these data with respect to the whole country population. 

 - Table 1: I'm assuming possible overlap between the "Covid-19" and the "Covid-19 Vaccine and Vaccination" subsets. This, of course, does not undermine the validity of the analysis in any sense, but it should be pointed out to the reader.

Is the work clearly and accurately presented and does it cite the current literature?

Partly

If applicable, is the statistical analysis and its interpretation appropriate?

Yes

Are all the source data underlying the results available to ensure full reproducibility?

No

Is the study design appropriate and is the work technically sound?

Yes

Are the conclusions drawn adequately supported by the results?

Yes

Are sufficient details of methods and analysis provided to allow replication by others?

Partly

Reviewer Expertise:

sentiment analysis; subjective well-being evaluation; statistical methods for causal inference

I confirm that I have read this submission and believe that I have an appropriate level of expertise to confirm that it is of an acceptable scientific standard, however I have significant reservations, as outlined above.

# Response

**Published:** 2023-11-19

**Authors:** Viskasari Kalanjati

**Affiliations:** Airlangga University, Indonesia

The paper offers a sentiment and opinion analysis about the reactions to Covid-19 pandemic, vaccination strategies and measures of social restrictions in Indonesia. The data source is a collection of Twitter messages posted between January 2020 and August 2021.

 The topic is interesting, its relevance is well argued and the results are properly presented and discussed.

**Answer**: We highly appreciate your comment.

 My main remarks concern the opportunity of providing more detailed information about the methodology the Authors apply in the study.

 The unavailability of the data (due to privacy constraints) makes it necessary to be clear about the analytical procedures illustrated in Fig.1, in order to ensure the replicability of the study, albeit with different data and to avoid, as far as possible, any "black box" effect.

 In particular:

 a) I assume that the data were labeled into the categories indicated in Table 1 in the downloading stage. Then they were further classified into "classes, which were tweet exposure and engagement analysis" (p.3): how was this classification stage performed?

 —

**Answer**: Thank you for the question. We try to clarify the explanation of the stages of analysis in the manuscript. Please find it in the revised manuscript, in the method chapter.

 b) before classifying data, a cluster analysis was applied using IndoBERT model. What is the role of cluster analysis in the study? Is there any relationship between cluster analysis and classification stages? Or, alternatively, is cluster analysis only aimed at visual inspection of data (in this case, it should be described before the classification stage, in order to avoid misunderstanding)? 

 —

**Answer**: We highly appreciate your question. We have clarified the explanation of the method. Please find it in the revised manuscript.

 We reworded the term of cluster analysis. We used IndoBERT model to label all cleaned data within the categories of positive, neutral, and negative sentiments. 

 IndoBERT (Bidirectional Encoder Representations from Transformers) constitutes a self-contained Deep Learning model designed for Natural Language Processing (NLP), inspired by the Transformer model. Each output element in this model is intricately connected to every input element, with dynamically computed weights based on inter-element relationships. BERT is formulated to aid computers in comprehending the ambiguous meaning of language within a text by utilizing the surrounding text to establish context.

 IndoBERT represents the Indonesian iteration of the BERT model, a Deep Learning model tailored for Natural Language Processing (NLP). This model is trained using over 220 million words in the Indonesian language. BERT is engineered to assist computers in understanding the ambiguous meaning of language within a text by leveraging the surrounding text to construct context, thereby obtaining improved word weighting values.

 In this study, IndoBERT is employed for the tokenization process of words before engaging in the classification task. Following the successful tokenization of the Indonesian language by IndoBERT, the data proceeds directly to the classifier layer to discern patterns in word tendencies, classifying them into three categories: positive, neutral, and negative.

 c) please, provide details on how the sentiment analysis is performed: how is a tweet evaluated as positive, negative or neutral? At p.5 (point 2) the manual classification of a 3000-texts-size random sample is described and indicated as a training set for the IndoBERT model. How does the IndoBERT model work? Is the 75% accuracy rate the result of a test by the Authors themselves?

**Answer**: Thank you for the question.  

 The training and testing processes within the IndoBERT model depend on a dataset prepared by the researcher, comprising 3000 randomly selected text data. The entirety of this process involves the researcher labeling the data into three classes. Upon completion of the labeling process, the data is subsequently partitioned into training and testing sets at an 80%:20% ratio. The training data is utilized by the IndoBERT model to discern patterns within words indicative of their tendency to fall into one of three categories: positive, neutral, or negative.

 After the model completes its training process, the subsequent step involves testing the data. Specifically, the model attempts to predict text data using the test data and compares these predictions with the labels assigned by the researcher. This comparative analysis aims to demonstrate the model's performance in classifying text sentiment. The results of this testing reveal an accuracy value of 75%, evaluated against the labels predicted by the model and those designated by the researcher. This accuracy value signifies the model's successful acquisition of patterns in the Indonesian language and its proficient performance in sentiment classification.

 d) p.2, point 2: the description of the content of positive, negative and neutral tweets is accompanied by a sort of opinion analysis: is it the result of a reading of the training sample or has an opinion analysis been actually extended to the whole dataset?

 —

**Answer**: Thank you for the question, The opinion analysis has been applied to the whole dataset.

 e) despite the low incidence of fake tweets, the Authors invite not to undervalue the impact of fake news. Is there any way for identifying a fake news diffusion pattern, following retweets, replies and "like"s, as these seem to be available pieces of information (see p.6)?

 —

**Answer**: Thank you for the question. This is an interesting topic to learn more about the diffusion pattern of circulating fake news, that necessarily will be useful for combating the spread of fake news. For further research, the idea is to capture all the metadata of users who interacted with the main post that the model has predicted as fake news by crawling all replies, likes, and retweets. This enables us to track how far the influences of the first posts are. It is also interesting for further study about the development of automated reply that notifies the main post audience that this Tweet has been classified as fake news, and provides a valid source of information

 In order not to move the focus of the paper from a public health to an artificial intelligence one, going into a more detailed description of the methodology may require a methodological appendix, where some examples could also be provided.

**Answer**: Thank you for the suggestion. We highly appreciate it. Since this journal applies an open review system whereby all readers can access the communication between authors and reviewers, details of the methods can easily be found through this communication. Therefore, we do not add the appendix.

 Minor remarks:

 - as usual, when a sentiment or opinion analysis is performed via social network sites data, some words should be spent to comment the lack of representativeness of these data with respect to the whole country population.

**Answer**: Thank you for the suggestion. We are pleased to accommodate it.

 - Table 1: I'm assuming possible overlap between the "Covid-19" and the "Covid-19 Vaccine and Vaccination" subsets. This, of course, does not undermine the validity of the analysis in any sense, but it should be pointed out to the reader.

**Answer**: Thank you for the suggestion. We are pleased to accommodate it.
